# Accelerated Electro-Fermentation of Acetoin in *Escherichia coli* by Identifying Physiological Limitations of the Electron Transfer Kinetics and the Central Metabolism

**DOI:** 10.3390/microorganisms8111843

**Published:** 2020-11-23

**Authors:** Sebastian Beblawy, Laura-Alina Philipp, Johannes Gescher

**Affiliations:** 1 Department of Applied Biology, Institute for Applied Biosciences, Karlsruhe Institute of Technology, Fritz-Haber-Weg 2, 76131 Karlsruhe, Germany; sebastian.beblawy@uni-tuebingen.de (S.B.); laura-alina.philipp@kit.edu (L.-A.P.); 2 Institute for Biological Interfaces, Karlsruhe Institute of Technology (KIT), Hermann-von-Helmholtz-Platz 1, 76344 Eggenstein-Leopoldshafen, Germany

**Keywords:** electro-fermentation, acetoin, methylene blue, bulk chemicals, metabolic engineering, *Escherichia coli*

## Abstract

Anode-assisted fermentations offer the benefit of an anoxic fermentation routine that can be applied to produce end-products with an oxidation state independent from the substrate. The whole cell biocatalyst transfers the surplus of electrons to an electrode that can be used as a non-depletable electron acceptor. So far, anode-assisted fermentations were shown to provide high carbon efficiencies but low space-time yields. This study aimed at increasing space-time yields of an *Escherichia coli*-based anode-assisted fermentation of glucose to acetoin. The experiments build on an obligate respiratory strain, that was advanced using selective adaptation and targeted strain development. Several transfers under respiratory conditions led to point mutations in the *pfl*, *aceF* and *rpoC* gene. These mutations increased anoxic growth by three-fold. Furthermore, overexpression of genes encoding a synthetic electron transport chain to methylene blue increased the electron transfer rate by 2.45-fold. Overall, these measures and a medium optimization increased the space-time yield in an electrode-assisted fermentation by 3.6-fold.

## 1. Introduction

Sustainable bioproduction will be the key to reaching the goal of a socio-economic transition into a bioeconomy. A low energy gain for the organisms and consequently a high catabolic production rate accompanied by low anabolic substrate conversion is favorable for fermentation-based biotechnological processes. This can be achieved under anoxic conditions. However, anoxic fermentations for instance the conversion of glucose to lactic acid or ethanol and carbon dioxide come with the boundary condition that the products must have the same oxidation state as the substrate. A more oxidized end-product demands the addition of an electron acceptor as oxygen. An elegant trade-off between both strategies was presented by Causey and colleagues. In this study, they disconnected oxygen reduction from energy generation by deleting parts of the membrane-bound ATPase (*ΔatpFH*) [1]. Although this concept solves the problem of an increasing biomass production rate, it cannot prevent the dissipation of a not negligible amount of energy in the form of heat.

A possible further solution could be to couple a respiratory process with an infinitely available electron acceptor in an electrode-assisted fermentation [2,3,4]. Here, a solid-state anode of a bioelectrochemical system is the electron acceptor for the microorganisms. This electron acceptor can be poised to a defined potential which will also determine the kinetics of the fermentation process to a certain extent [5]. Moreover, the lower the potential at which the electrons are transferred to the anode the more energy they carry. This energy can for instance be used via the concept of a microbial electrolysis cell in which the electrons are used to produce hydrogen on the cathode side. Using a limited amount of further energy (cell voltages typically between 0.6 V and 0.9 V), which is roughly 50% compared to conventional water electrolyzers (typically operating at 1.6 to 1.8 V), the microbially produced current can be used on the cathode side for the production of hydrogen [6,7]. Moreover, an electrode-assisted fermentation can be conducted in a biofilm reactor which offers the benefit of continuous production processes using a natural retentostat, which makes it easy to separate the end-product from the substrate and the biocatalyst [8]. 

The direct respiratory interaction of microbes with anodes is restricted to a certain physiological class of microorganisms. These microbes contain an extended respiratory chain that consists usually out of *c*-type cytochrome proteins. One of the best-understood model organisms for anode respiration is *Shewanella oneidensis*, a *γ*-proteobacterium. *S. oneidensis* uses a tetraheme *c*-type cytochrome called CymA to transfer electrons from the menaquinone pool into the periplasm [9]. The two electron shuttling proteins Stc and FccA transfer the electrons through the periplasm and onto a trimeric complex in the outer membrane that consists of one *c*-type cytochrome on the periplasmic site and the cell surface, respectively, which are connected by a β-barrel protein [10,11]. The two outer membrane decaheme cytochromes MtrC and OmcA are the terminal reductases of the organism and possibly form a loosely attached complex [12,13,14].

**Figure 1:**
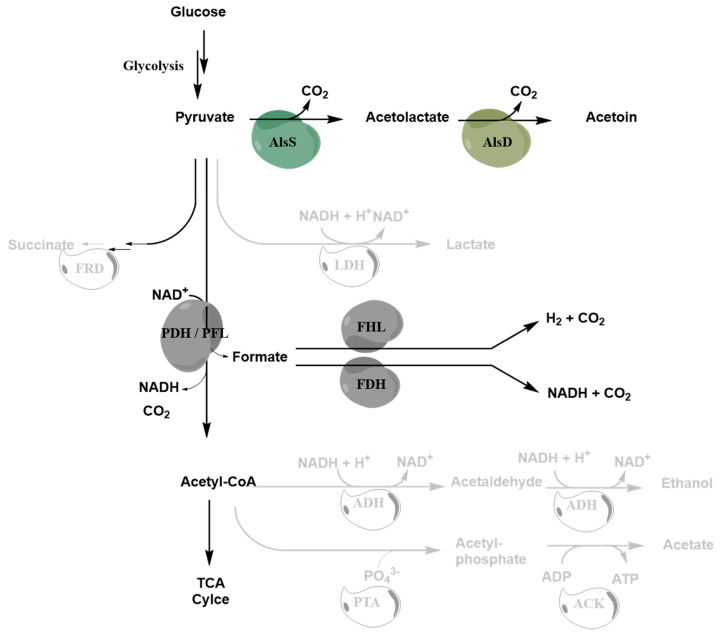
Overview of the modifications introduced by Förster et al. (2017) to the central metabolism of *E. coli* to facilitate the respiration dependent conversion of Glucose to Acetoin (JG991) [22]. Green shapes represent the heterologously expressed enzymes AlsS and AlsD, grey shapes are native enzymes of *E. coli* and hollow shapes and faded reactions indicate pathways that are blocked by gene deletions.

A disadvantage of *S. oneidensis* is, that the organism has a rather restricted spectrum of carbon sources it can use under anoxic conditions and that it forms only thin biofilms on electrode surfaces. Therefore, several research groups tried to develop processes using different biocatalysts. One of these organisms is *Pseudomonas putida* that could be coupled to an anode as the electron acceptor for the production of 2-ketogluconate from glucose [15,16]. Electron transfer to the anode was possible by using different electron shuttles which were characterized by redox potentials above 0.207 V against a standard hydrogen electrode. With a similar strategy, *Corynebacterium glutamicum* was applied in an anode-assisted fermentation of lysine using ferricyanide as a redox mediator [17]. Another strategy to connect biocatalysts with an anode as the electron acceptor is the transplantation of an extracellular electron transport chain into a recipient biocatalyst strain, to enable either direct electron transfer or accelerate electron transfer using electron shuttles [18,19,20,21]. Förster et al. [22] developed an acetoin producing *E. coli* strain by deleting crucial genes of the mixed acid fermentation pathway (*adhE*, *ldhA*, *frdA-D* and *ack-pta*) and expressing the acetolactate synthase (*alsS)* and acetolactate decarboxylase (*alsD*) of *Bacillus subtilis* (Figure 1). In this process a fermentation deficiency was achieved, rendering the metabolism under anoxic conditions dependent on respiratory electron acceptors such as nitrate or DMSO. Furthermore, they inserted the necessary elements of the electron transport chain of *S. oneidensis* to transfer electrons into the periplasm. Electron transfer to the anode was achieved by the aid of the membrane-permeable electron shuttle methylene blue that can be reduced by the cytochromes in the periplasm and reoxidized by interaction with the anode. Still, the deletion of key genes for fermentation pathways alone resulted in a pronounced decrease in the growth rate with several anoxic electron acceptors. Nevertheless, it was possible to achieve with this strain an electrode-assisted acetoin production process, that was characterized by very high carbon efficiencies but unfortunately low space-time-yields. 

This study reports on several measures that were taken to increase the fitness of the strain under anoxic conditions and to increase the methylene blue reduction rates. We discovered—using adaptation experiments and resequencing of the resulting strain—mutations that increased the fitness of the strain under anoxic respiratory conditions. The genes identified in this experiment hint towards an anabolically constraint metabolism. Moreover, overexpressing key-genes for the electron transfer process we increased the methylene blue reduction rate of the organisms. In bioelectrochemical experiments, we demonstrate the success of the approach by increased production rates.

## 2. Materials and Methods

### 2.1. Vector and Strain Construction

The copy number of the *cymA* and *stc* gene in the *E. coli* strain JG1397 (Table 1) was increased by cloning the two genes behind the *alsSD* genes in the pMAL plasmid derived from Förster et al. [22] (Table 2). The *cymA* and *stc* genes were first codon-optimized for use in *E. coli* and amplified using Primer 1 and 2 (Table 3, Appendix A Sequence A4 and A5). Amplification also led to the elongation of the PCR fragment by homologous regions to the pMAL_*alsSD* plasmid. After cleaving this plasmid with SalI (New England Biolabs) the PCR fragment was inserted via isothermal DNA assembly [23]. The resulting plasmid was sequenced and transformed into JG1352 together with pEC86.

### 2.2. Cell Cultivation

Standard cultivation of *E. coli* was conducted at 37 °C and 150 rpm in LB medium (1% *w/v* yeast extract, 0.5% *w*/*v* NaCl, 0.5% *w*/*v* peptone) supplemented with antibiotics for plasmid maintenance if necessary. Growth experiments were conducted in phosphate-buffered medium (137 mmol/L NaCl, 2.7 mmol/L KCl, 10 mmol/L Na_2_HPO_4_, 1.76 mmol/L KH_2_PO_4_, 9 mmol/L (NH_4_)_2_SO_4_, 1 mmol/L MgSO_4_, 0.1 mmol/L CaCl_2_, 10 mL/L trace element solution (100×: 0.27 mmol/L CoCl_2_, 0.02 mmol/L CuSO_4_, 5.66 mmol/L H_3_BO_3_, 0.24 mmol/L FeCl_2_, 6.72 mmol/L Na_2_-EDTA, 0.13 mmol/L MnSO_4_, 0.22 mmol/L Na_2_MoO_4_, 0.13 mmol/L Na_2_SeO_4_, 1 mmol/L NaCl, 0.5 mmol/L NiCl, 0.16 mmol/L ZnSO_4_), 1 g/L casein hydrolysate or yeast extract, 14.8 µmol/L thiamine, 20 mmol/L glucose) if not stated otherwise. Anoxic growth experiments were conducted in sealed bottles with 40 mmol/L NO_3_^−^ or DMSO added to the medium. The medium was boiled before use and sparged with N_2_ gas. The initial OD_600_ for all growth experiments was 0.05. If necessary, kanamycin (10 µg/mL), ampicillin (100 µg/mL), and chloramphenicol (30 µg/mL) were added to the medium. pMAL_*alsSD* or pMAL_*alsSD*_*cymA*_*stc* were induced using 50 µmol/L IPTG. For the induction of the *cymA*-*mtrA* locus and the *stc* locus, the culture was supplemented with 0.43 µmol/L anhydrotetracycline and 1 mmol/L arabinose. Cell density was monitored photometrically using a SPECTRONIC Genesys 20 spectrophotometer (Thermo Fisher Scientific, Waltham, MA, USA) or an Ultrospec 10 Cell Density Meter (BioChrom, Holliston, MA, USA).

### 2.3. Adaptive Laboratory Evolution (ALE)

ALE selection for an accelerated anaerobic metabolism with DMSO was performed in Hungate tubes containing 10 mL MOPS-buffered medium (137 mmol/L NaCl, 2.7 mmol/L KCl, 15 mmol/L MOPS, 9 mmol/L (NH_4_)_2_SO_4_, 1 mmol/L MgSO_4_, 0.1 mmol/L CaCl_2_, 10 mL/L trace element solution, 1 g/L casein hydrolysate or yeast extract). The medium was supplemented with 20 mmol/L Glucose, 40 mmol/L DMSO, 100 µmol/L methylene blue, and 14.8 µmol/L thiamine. The tubes were then inoculated with JG991 to an initial OD_600_ = 0.05. These cultures were incubated at 30 °C and transferred into new tubes when the suspension became visibly turbid (OD_600_ ≈ 0.3). For this purpose, the optical density was determined in all approaches at the beginning and end of each transfer and the number of generations was calculated ($n=log_{2}\;N-\;log_{2}\;N_{0}$, *n* = number of generations, *N* = cell number). The next set of culture tubes was then inoculated with 1 mL culture of the tube with the highest number of cell divisions. 

### 2.4. Methylene Blue Reduction Assay

The methylene blue reduction rate was determined in an anoxic cell suspension assay (OD_600_ = 6) conducted in an anoxic chamber (Coy Laboratory Products, Grass Lake, MI, USA) with an N_2_/H_2_ atmosphere (98%/2%). Cells were pre-grown under anoxic conditions in M9 medium (9.2 mmol/L NaCl, 21.2 mmol/L KH_2_PO_4_, 18.7 mmol/L NH_4_Cl, 47.8 mmol/L Na_4_HPO_4_, 9 mmol/L, MgSO_4_, 0.1 mmol/L CaCl_2_, 10 mL/L trace element solution (100×: 0.27 mmol/L CoCl_2_, 0.02 mmol/L CuSO_4_, 5.66 mmol/L H_3_BO_3_, 0.24 mmol/L FeCl_2_, 6.72 mmol/L Na_2_-EDTA, 0.13 mmol/L MnSO_4_, 0.22 mmol/L Na_2_MoO_4_, 0.13 mmol/L Na_2_SeO_4_, 1 mmol/L NaCl, 0.5 mmol/L NiCl, 0.16 mmol/L ZnSO_4_), 1 g/L casein hydrolysate, 14.8 µmol/L thiamine) with 50 mmol/L DMSO as sole electron acceptor and 0.5% *w*/*v* glycerol as carbon source. Antibiotics and, if necessary, IPTG, AHT, and arabinose were added as described above. The expression of the heterologous genes was induced at an OD_600_ of 0.3. Cells were harvested and washed twice before the start of the assay. Cells were then diluted in 50 µL media without an electron acceptor into a microtiter plate to an OD_600_ of 12. The suspension was incubated for 30 min to remove any residual oxygen. To start the reduction assay, 50 µL methylene blue with a concentration of 546 µmol/L were added simultaneously to each well with a dispenser tool (Infinite 200 PRO, Tecan, Männedorf, Switzerland). Absorption was measured at 620 nm using the Infinite 200 PRO spectrometer (Tecan, Männedorf, Switzerland). The assay was typically completed within 180 s.

### 2.5. Preparation of Nucleic Acids

The preparation of plasmids and chromosomal DNA was carried out using 2 mL of an aerobic culture in LB medium. The plasmids were isolated using the Wizard Plus Miniprep DNA Purification System (Promega, Madison, WI, USA). The Wizard Genomic DNA Purification Kit (Promega, Madison, WI, USA) was used to isolate chromosomal DNA. Deviating from the protocol, the isolated DNA was rehydrated in the last step in 75 µL Tris/HCl buffer (15 mmol/L). Nucleic acids were quantified using a NanoDrop 2000 spectrophotometer (Thermo Fisher Scientific, Waltham, MA, USA). Chromosomal DNA was further quantified using the QBit dsDNA BR Assay Kit (Thermo Fisher Scientific, Waltham, MA, USA).

### 2.6. Sequencing

Plasmid DNA was sequenced using the Mix2SeqKit (Eurofins Genomics, Ebersberg, Germany). The genomes of JG1295 and JG991 were resequenced using an Illumina MiSeq with v2 chemistry (Illumina, San Diego, CA, USA) with 2 × 250 bp reads for the variant analysis. The library was created using the NEBNext^®^ Ultra™ II FS DNA kit. Illumina sequencing was carried out by IMGM Laboratories GmbH (Martinsried, Germany). 

### 2.7. Bioinformatic Analysis

The analysis of sequencing data was conducted using the CLC Genomics Workbench 11 (Qiagen, Venlo, The Netherlands). The workflow for NGS data contained: merging forward and reverse reads, a quality trim (*p* = 0.05), and mapping reads to the reference genome. The consensus sequence of the reference genome resequencing has been extracted by majority vote. This consensus sequence has been used as the reference for a variant analysis of the strain obtained from the ALE experiment. Variants with a fidelity >90% and a coverage of 20× were regarded as significant.

### 2.8. Electro-Fermentation

**Figure 2:**
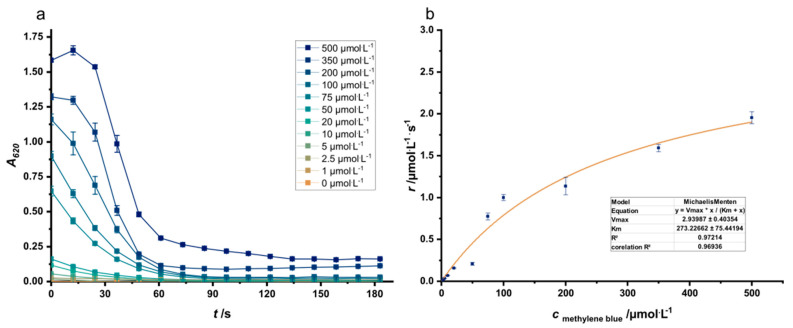
Reduction kinetics of methylene blue for Strain JG991 (**a**). Assays have been conducted in PBS minimal medium with an OD_600_ = 6. The highest reduction rates have been fitted to a Michaelis-Menten model (**b**).

In brief, the bioelectrochemical system had a volume of 23 mL. The working electrode was a graphite felt (40 cm^2^ surface, GFD 2.5, Sigracell, Germany), and the counter electrode a platinum mesh (1.25 cm^2^, chemPUR, Karlsruhe, Germany). An Ag/AgCl electrode (Xylem group, Germany) was used as a reference. The anode potential was poised to 0 mV against Ag/AgCl. The detailed procedure including schematics of the reactor system can be found in Förster et al. [22]. Differing from that, a MOPS buffered medium was used supplemented with 0.1% yeast extract and 10 or 50 mmol/L Glucose. The pH value of 7.2 was maintained using a pH-controller (Hach Lange, Düsseldorf, Germany) feeding a 0.5 mmol/L NaOH solution. The electrical current was recorded for 8 h. Of note, the doubling time of the organisms. under these conditions will be at least 20 h (Figure 2). 

### 2.9. Analytical Methods

The chemical analysis of the growth experiments and cell suspension assays was conducted via HPLC (Ultimate 3000 DAD and RI-Detector, Thermo Scientific, Waltham, MA, USA) with an Aminex HPX 87-H column (Bio-Rad, Hercules, CA, USA) at 60 °C with 5 mmol/L H_2_SO_4_ as the mobile phase. Acetoin was also quantified using the Voges-Proskauer reaction as described by Förster et al. [22].

### 2.10. Calculation of Evaluation Parameters

The electro-fermentation experiments have been evaluated according to the average current density within the 8 h time frame of the experiment (j), coulombic efficiency (CE), yield (Y), and productivity (Q). The parameters have been calculated following these formulas:

(1) $$j=\frac{\int_{t_{0}}^{t_{8}}I}{t_{8}}\;\mathrm{CE}=\frac{\int_{t_{0}}^{t_{8}}I}{{∆}_{c}\cdot V_{r}\cdot z_{e}\cdot \epsilon \cdot N_{A}}\;Y=\frac{∆c_{Ace}}{∆c_{Gluc}}\;Q=\frac{∆c_{Ace}\;\cdot M_{Ace}}{∆t}$$

*I*: electrical current, *A*: electrode surface, t: time, *V_r_*: reactor volume, *z_e_*: electrons per reaction, *c*: concentration, *ϵ*: elementary charge, *M*: Molar Mass, *Ace*: Acetoin, *Gluc*: Glucose, *N_A_*: Avogadro constant.

## 3. Results and Discussion

In a previous study, we established the production of acetoin from glucose in an electrode-assisted fermentation [22]. The addition of methylene blue was necessary to allow electron transfer from the periplasm to the anode surface. While the carbon efficiency set a new benchmark, the space-time yield was rather low compared to oxic processes. Hence, the initial aim of this study was to elucidate the theoretically achievable conversion rate based solely on the kinetics of methylene blue reduction by the strain. Therefore, *K*_M_ and *V*_max_ of methylene blue reduction were determined in a microtiter-based reduction assay. The measured methylene blue reduction rate for the *K*_M_ value of 273 µmol/L was 1.4 µmol/L/s. In comparison, the rate for 50 µmol/L was 0.45 µmol/L/s (Figure 2). This value is equivalent to a theoretical current density of 498.35 mA/m^2^ under conditions applied in the experiment of Förster et al. [22]. In said experiment, however, only 115.8 ± 6.7 mA/m^2^ were measured. Hence, if the reduction rate of the production strain in an electro-fermentation setup could converge to the rates of this idealized reduction assay, an increase in the metabolic activity of up to 4.3-fold could be achieved. For a methylene blue concentration of 273 µmol/L which equals the *K*_M_ value of the cellular system an increase of up to 13.9-fold might be possible. Still, this theoretical transformation of methylene blue reduction kinetics into current densities does not account for a loss of mediator over time in the BES (bioelectrochemical system), it also neglects possible mass transport limitations with regards to substrate uptake. Moreover, the rather fast initial methylene blue reduction kinetics will be hampered by the growth inhibiting effect of this electron shuttle that can also pass the inner membrane [26,27]. Still, the comparison of the data achieved from electro-fermentation experiments with these theoretical values reveals to which account the mentioned factors indeed limit the overall process and give a clear maximum for the achievable current density with the initial strain’s physiology.

### 3.1. Adaptation to Improved Anoxic Respiration

Previous experiments revealed that the deletion of all fermentation pathways in the developed production strain led to a severe limitation of growth also under anoxic respiratory conditions. The physiological reason for this remained at least partly enigmatic. The deletion of *ack/pta* will lead to a reduced production of ATP that will influence the growth rate and yield. Interestingly, when strains with an increasing number of deletions in fermentation pathways were compared, it was observed that the growth rate was already decreased to 17% of the initial level after the first two deletions steps (*frd* and *adh* gene). Further, the deletion of *ldh* lowered the growth rate to 8% of the initial level and the loss of *ack/pta* to 5%. In contrast, the deletions of *frd*, *adh*, and *ldh* combined reduced the biomass yield to 50% of the initial level, while the additional deletion of the *pta/ack* locus led to 15% of the initial value [22]. Albeit, the growth and yield data suggest that the deletion of NADH reoxidation pathways seems to have a general dysfunctional central metabolism as consequence. Hence, we aimed for an adaptation experiment and hypothesized that consecutive transfers on the anoxic medium would select for mutations that lead to a more balanced central metabolism and consequently faster growth.

**Figure A1:**
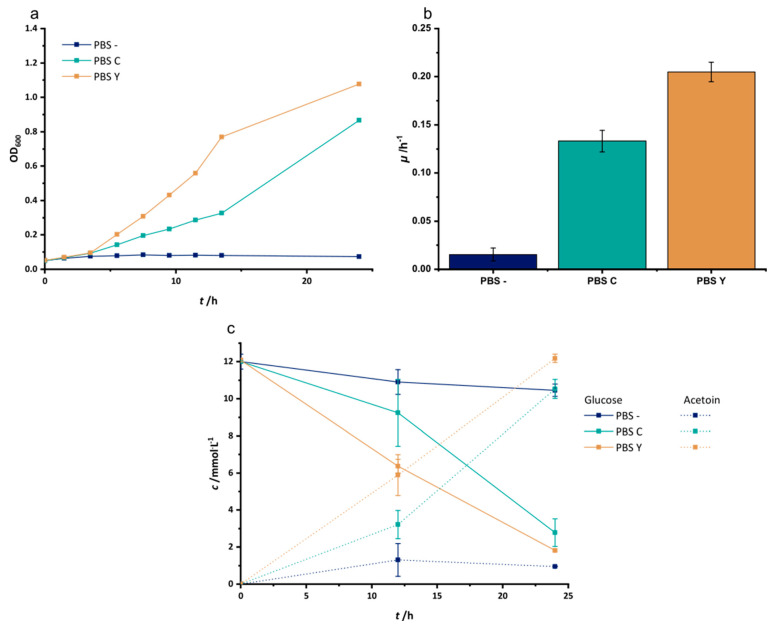
Influence of media supplementation on the metabolic rate of JG991. (**a**) shows the anaerobic growth of JG991 in PBS minimal medium with 40 mmol/L NO_3_^−^ and 12 mmol/L Glucose supplemented with 0.1% casein hydrolysate (PBS C) or 0.1% yeast extract (PBS Y). PBS—is without supplementation. The growth rates are represented in (**b**). The analysis of substrate consumption and product formation is represented in (**c**).

**Figure A2:**
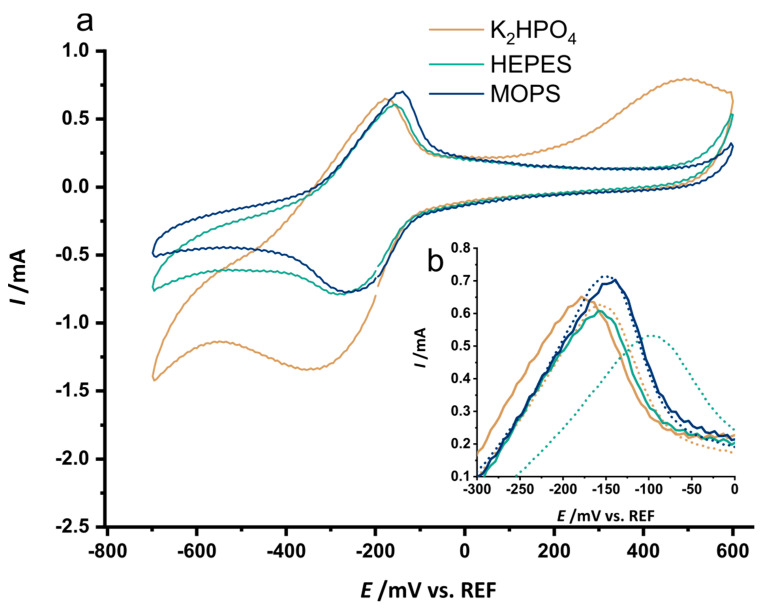
(**a**) Cyclic-Voltammetry of 50 µmol/L methylene blue in different buffer components as base for the electro-fermentation medium at a scan rate of 20 mV/s. yellow—K_2_HPO_4_, teal—HEPES, blue—MOPS, each 15 mmol/L. (**b**) Peak currents for the methylene blue oxidation in differently buffered media compositions from A (compact lines) and fully complemented media compositions including 20 mmol/L Glucose and 1 g/L yeast extract (dotted lines).

The medium for the selection experiment was altered compared to our previous study since growth experiments revealed that the addition of yeast extract instead of casamino acids can increase anoxic growth by the strain (Appendix A Figure A1). Moreover, we aimed for optimizing the medium concerning the later application in a bioelectrochemical system. Hence, we determined the effect of either phosphate-, HEPES- or MOPS-buffer on the electrochemical interaction of methylene blue with the electrode using cyclovoltammetry. The experiments revealed that the highest current could be observed using MOPS-buffer, while the application of HEPES and phosphate-buffer led to 25% and 12% reduced peak currents (Appendix A Figure A2a). Moreover, the addition of yeast extract to the medium did not influence the methylene blue redox reaction in the conducted cyclovoltammetry experiments (Appendix A Figure A2b). 

**Figure 3:**
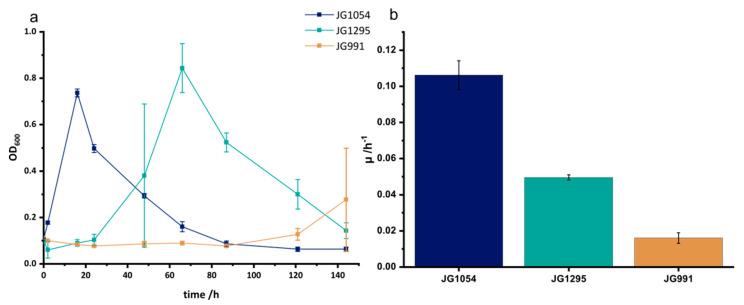
Anaerobic growth in MOPS buffered medium supplemented with 40 mmol/L DMSO, 20 mmol/L glucose, and 0.1% yeast extract. (**a**) Growth curve for the previous production strain (JG991) and the newly adapted strain (JG1295) in comparison to a reference strain without modifications in the mixed acid fermentation pathway (JG1054). The growth rates for this experiment are represented in (**b**).

**Figure 4:**
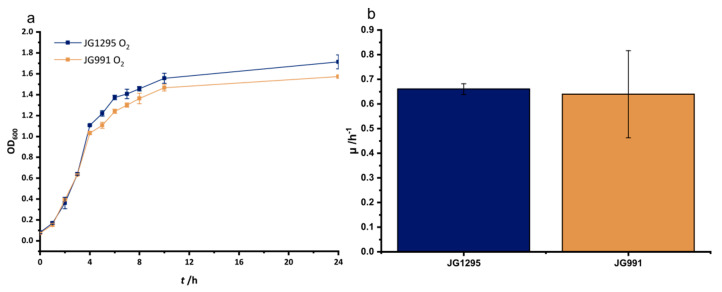
Aerobic growth in MOPS- Medium supplemented with 20 mmol/L glucose and 0.1% yeast extract. (**a**) Displayed are growth curves for the previous production strain (JG991) and the newly adapted strain (JG1295). The growth rates for this experiment are represented in (**b**).

With this optimized medium, we started the selection experiment using DMSO as the electron acceptor and glucose as the electron donor. DMSO was chosen as its reduction is dependent on menaquinol which is also the electron donor for the heterologously produced CymA from *S. oneidensis*. Production of the latter is key for the introduced synthetic electron transport chain to methylene blue. After 16 transfers, the growth rate increased 3-fold from 0.016 ± 0.003 for the progenitor strain JG991 to 0.050 ± 0.001 for the developed strain, which will be referred to as JG1295 (Figure 3a,b). Of note, we continued the experiment for 7 further transfers but could not observe an increase in growth past transfer 16. It is further notable, that the lag phase has been reduced from approximately 100 h (JG991) to less than 24 h (JG1295). Still, an *E. coli* control strain that is not fermentation deficient shows no apparent lag phase and grows twice as fast as JG1295. Overall, the conducted adaptation reduced the gap in growth rate between control and adapted strain from 6-fold to 2-fold. Of note, the observed effect is specific for anaerobic growth since a control experiment under oxic conditions did not reveal a statistically significant difference between the growth rates of strain JG991 and JG1295 (Figure 4). 

### 3.2. The Adaptation to Growth on DMSO Is Based on Three Point Mutations

We resequenced the genome of the strain to correlate the phenotype of the organism to specific changes in the genome sequence. The analysis revealed three variants with frequencies above 90% and a coverage of at least 20× (Table 4). Two mutations were found in genes encoding enzymes of the central metabolism. A stop codon was introduced in the gene for the pyruvate-formate-lyase (*pfl*) by replacing amino acid (AA) 431 (glutamate). A second point mutation in the gene of the dihydrolipoamide acetyltransferase subunit (*aceF*) of the pyruvate dehydrogenase replaces aspartate 430 by glycine. Moreover, the gene for the RNA polymerase *rpoC* was modified by replacing proline 359 by leucine (AA359). 

As the mutation in *pflB* leads to an interruption of the translation after approximately half the gene, we have to assume that pyruvate will not be converted to acetyl-CoA and formate via this enzyme anymore. Hence, it is interesting to observe that a second mutation occurred in an enzyme that can also catalyze the conversion of pyruvate to acetyl-CoA. The point mutation in *aceF* cannot lead to an inactive enzyme as this would cause no or at least very slow growth under oxic conditions, which could not be observed in this study (Figure 4) [28]. The *aceF* gene encodes subunit E2 of the pyruvate dehydrogenase to which E1 and E3 bind non-covalently. E2 consists of three lipoyl-domains at the N-terminus, a peripheral subunit-binding domain, and a large C-terminal core-forming acetyl-transferase domain to which the here observed mutation is localized [29,30]. Although the pyruvate-formate-lyase is the major pyruvate converting enzyme under anoxic conditions, the expression levels of the pyruvate dehydrogenase do not seem to be negatively influenced by the absence of oxygen [31]. Still, the activity of the enzyme is regulated by the NADH sensitivity of the dihydrolipoamide acetyltransferase subunit and the prevailing higher NADH/NAD ratios under anoxic conditions [22,23,24,25,26,27,28,29,30,31,32,33,34]. In fact, screening for mutants with higher ethanol productivity revealed clones with point mutations in the *aceF* gene, which rendered the enzyme insensitive to NADH-inhibition. Still, these mutations (H322Y or E354K) occurred at different positions [31]. Nevertheless, the situation is different in the here studied strain and a selection for an NADH-insensitive enzyme is unlikely. The genes for the majority of enzymes that would consume acetyl-CoA in the wild type under anoxic conditions were deleted. As the citric acid cycle is also downregulated, it would be detrimental for the strain to produce higher concentrations of acetyl-CoA, as accumulating CoA-esters can be toxic to the cell [35]. Moreover, we know that the carbon yield under acetoin-producing conditions is not lower (see below) compared to the strain before ALE which suggests that the available pyruvate pool is at least stable. Additionally, the mutation in the *aceF*-gene does not lead to a growth disadvantage under oxic conditions, which would indicate a generally reduced enzymatic activity. Hence, we hypothesize that the mutations render the enzyme even more stringent regarding reduced activity under the cellular conditions during anoxic growth. The NADH to NAD ratio as well as the cellular pyruvate concentrations could be used as triggers to reduce the enzyme’s activity. The TCA cycle could still be delivered by necessary intermediates via the reduced acetyl-CoA pool and—probably more important—via the routes from pyruvate or phosphoenolpyruvate to oxaloacetate [36]. These three delivery routes will most likely be sufficient to support building block formation for biomass production under DMSO reducing conditions. Following this line of thought, the observed truncation and most probable deactivation of the pyruvate formate lyase would be a selective advantage as it simply stops the major acetyl-CoA producing reaction under anoxic conditions. The high pyruvate flux establishes in wild type *E. coli* an acetyl-CoA pool that is mainly deployed for energy production and redox balancing reactions [37,38]. As the mutant cannot catalyze these reactions anymore the high flux will not be relevant to sustain growth and—as described above—detrimental due to CoA-ester accumulation. While it is from a physiological viewpoint straight forward to hypothesize on the function of the *pfl* and *aceF* mutations it sems impossible to easily explain the potential impact of the *rpoC* mutation. RNA polymerase genes are often found to be targets for mutation in a multitude of screens and it is not always obvious how the particular mutation leads to the observed change in phenotype [39]. Nevertheless, we are not aware of a screen that would have revealed particularly this mutation before [40]. Overall, it is difficult to assess the influence of the individual point mutations as the two mutations in pyruvate converting enzymes will likely be interdependent regarding the growth stimulating effect. Moreover, it is not possible to easily assess the function of the *rpoC* mutation. The original strain grows poorly under anoxic respiratory conditions and partly forms flocks during growth. Hence, the mass limitations due to limited diffusion into the flocks will likely lead to an inhomogeneous transcriptome, which hampers comparison to the transcriptome of the strain after ALE.

### 3.3. The Selected Mutations Increase Productivity and Current Production in Bioelectrochemical Systems

**Figure 5:**
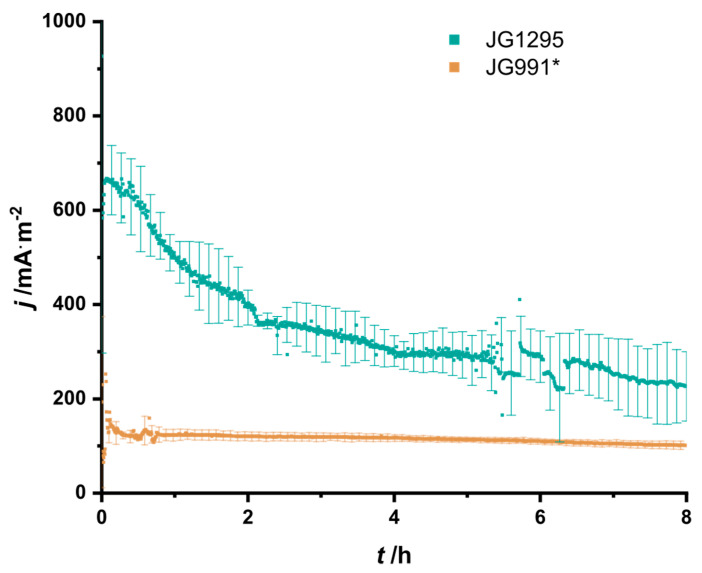
Chronoamperometric detection. Electro-fermentation in MOPS buffered medium supplemented with 10 mmol/L glucose and 0.1% yeast extract. Displayed is the current density over time for the previous production strain (JG991, yellow) and the newly adapted strain (JG1295, teal). * data originally published in [22] and added here as a reference for the performance of the newly adapted strain.

Next, we sought to transfer the experiments from the cell suspension assays to bioelectrochemical systems. Strain JG1295 was introduced into the systems with an initial OD_600_ = 6. The chronoamperometric measurement of the electro-fermentation at OD_600_ = 6 revealed an initial current density maximum of 667.7 ± 83.6 mA/m^2^ after 4 min followed by a slow decrease in current density (Figure 5). Overall, the process and strain optimization conducted in this study led to a 3.6-fold increase in productivity compared to the previous report by Förster et al. [22]. The coulombic efficiency remained with 88.1 ± 16.3% in the range on the previously observed high value of 84.4 ± 4.1%.

**Figure 6:**
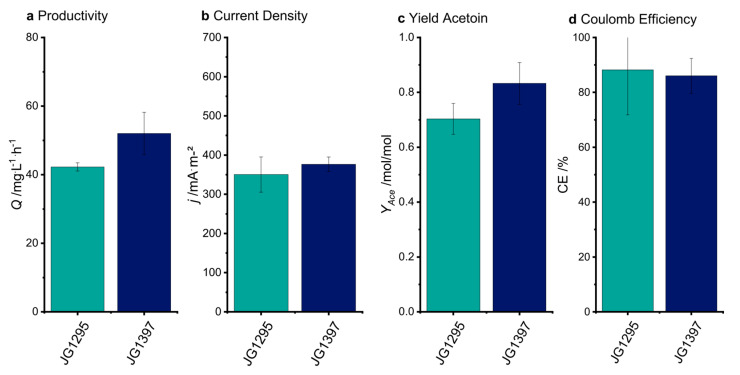
Evaluation of the electro-fermentation experiments for JG1295 and JG1397. All parameters are insignificant between the two strains. (**a**) Productivity (Q), (**b**) Average Current Density (j), (**c**) Yield Acetoin (Y_Ace_), (**d**) Coulomb Efficiency (CE).

**Figure A3:**
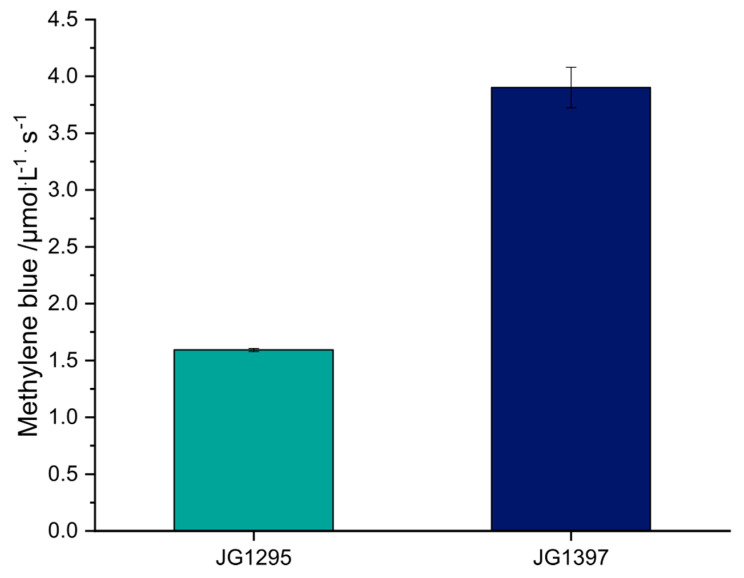
Reduction rates with 273 µmol/L methylene blue for JG1295 and JG1397 in a cell suspension of OD_600_ = 6.

The increase in current density agrees well with the observed acceleration in the growth rate on DMSO. Hence, it seems as if central metabolism and not the electron transfer kinetics limit the process since the conducted methylene blue reduction assays suggest that higher current densities could be achieved considering the maximum reduction rates obtained in cell suspension assays. To confirm that the limitation of the strain is most probably in central metabolism and not in electron transfer kinetics, we increased the expression rate of *cymA* and *stc* by introducing an additional plasmid-encoded copy of the two genes (strain JG1397). The plasmid also contained the *alsSD* genes for acetoin production. The introduction of the plasmid and expression of the genes increased the methylene blue reduction rate by 2.45-fold (Appendix A Figure A3). The experiments are consistent with previous results that reveal that *cymA* expression levels are constraining for extracellular electron transfer in *E. coli* and that the expression strength of genes encoding for cytochromes can have a crucial impact on electron transfer rates [41,42]. Still, the introduction of this strain to bioelectrochemical systems led to an insignificant increase in current density and acetoin yield compared to the progenitor strain JG1295 (Figure 6). This result bolsters the hypothesis that current density is limited by central metabolism rather than electron transfer kinetics.

## 4. Conclusions

Using a targeted and non-targeted evolution of a production strain for anode-assisted acetoin production from glucose, it was possible to raise the current density (3.6-fold) and consequently the productivity (3.6-fold) that can be achieved with this strain. 

This study started with establishing the characteristics of methylene blue reduction kinetics and a *K*_M_ value of 273 µmol/L was established for the whole-cell biocatalyst. Using this data and converting the measured reduction rates to a potentially achievable current density leads to a value of approximately 1.55 A/m^2^. While the initial current densities observed in the electro-fermentation experiment are only 2.3-fold lower as this value, the average current density throughout 8 h is 4.8-fold lower. This has the following implications: First, the development of the strain was successful as it is easily imaginable that the initially discussed mass transfer limitations of the BES cause the 2.3-fold difference in electron transfer rate. Second, this difference becomes more pronounced over longer periods is most probably due to the growth-inhibiting effect of methylene blue that is not interfering with the fast kinetics measured in cell suspension experiments. Hence, a further step towards a more stable current production would be to establish an *E. coli* strain that is more resilient towards higher methylene blue concentrations. Previous studies already revealed potential targets for a directed strain evolution approach. Apparently, the efflux pump AcrAB is necessary for *E. coli* to thrive in medium with higher methylene blue concentrations [43,44]. A defect in this efflux pump led to lacking growth already with 30 µmol/L methylene blue. The gene cluster is negatively regulated by AcrR. Nevertheless, our initial attempts to delete this repressor were so far not successful possibly due to a toxic effect of this mutation. 

Increasing space-time yields of electrode-assisted fermentations will be key for their future biotechnological implementation. The here conducted study provides a workflow for strain development and characterization of the capabilities of the applied BES. Further research will focus on accelerating glucose metabolism in the strain and aim at increasing the amount of active biocatalyst within the bioreactors by advancing reactor technology.

## Figures and Tables

**Table 1:** Strains used in this study.

No.	Based on	Relevant Genotype	Reference
JG22	*E. coli* DH5αZ1	*aciq*, *PN25-tetR*, *SpR*, *deoR*, *supE44*, *Δ(lacZYA* *argFV169)*, *Phi80* *lacZΔM15*	[24]
JG1054	JG22	pMAL *alsSD*	this study
JG991	JG22	*Δ(napC-F)*, *ΔfrdA-D::(Ptet cymA mtrA, mtrB)*, *ΔgalK*, *ΔadhE*, *idh::PAra stc*, *ΔldhA::cscRAKB*, *Δpta ackA::galK* pMAL *alsSD* pEC86	[22]
JG1295	JG991	*pfl^−^*, aceF*, *rpoC^−^*	this study
JG1352	JG1295	*Δ(napC-F)*, *ΔfrdA-D::(Ptet cymA mtrA, mtrB)*, *ΔgalK*, *ΔadhE*, *idh::PAra stc*, *pfl^−^*, aceF*, *rpoC^−^* *ΔldhA::cscRAKB*, *Δpta ackA::galK*	this study
JG1397	JG1352	pMAL *alsSD_cymA_stc* pEC86	this study

**Table 2:** Vectors used in this study.

Name	Relevant Genotype	Reference
pMAL *alsSD*	*bla*, P_Tac_*alsSD*	[22]
pMAL *alsSD_cymA_stc*	*bla*, P_Tac_*alsSD*, *cymA*, *stc*	this study
pEC86	*cat*, *P_Tet_ ccmABCDEFGH*	[25]

**Table 3:** Oligonucleotides used in this study.

No.	Sequence
1	CCCTTTTAGCAGGGCTTTCTGGAAGGAGATATACATACCATGAACTG
2	AAACGACGGCCAGTGCCAAGCTTGCCTGCAGGTTATTTTTTCAGAACAGATGCGC

**Table 4:** Variant analysis of JG1295 compared to JG991. Represented are variants with a frequency of above 90% and a coverage of 20×.

Base	Gene	Type	Mutation	Effect	Fidelity
4320496	*pflB*	SNV	G → A	Gln to Stop (AA431)	112/112 (100%)
3009449	*rpoC*	SNV	T → C	Pro to Leu (AA359)	155/156 (99.4%)
3593620	*aceF*	SNV	A → G	Asp to Gly (AA430)	136/138 (98.6%)

## Appendix A

**Sequence A4: Codon-optimized sequence of *cymA*.**

ATGAACTGGCGTGCACTGTTCAAACCGAGCGCAAAATACAGCATCCTGGCTCTGTTAGTTGTGGGTATCGTTATCGGCGTGGTTGGTTACTTCGCGACCCAGCAGACCCTGCACGCTACCTCTACCGATGCATTCTGCATGTCATGCCACAGTAACCACTCTCTGAAAAACGAAGTTCTGGCATCTGCGCACGGTGGAGGCAAAGCAGGTGTTACCGTTCAGTGCCAGGACTGCCACCTGCCGCACGGTCCGGTTGACTACCTGATCAAGAAAATCATTGTTAGTAAAGACCTGTACGGTTTCCTGACCATCGACGGTTTCAACACCCAGGCCTGGCTGGACGAAAACCGTAAAGAACAGGCAGACAAAGCGCTGGCATACTTCCGTGGTAACGACTCAGCTAACTGCCAGCACTGCCACACCCGTATCTACGAAAACCAGCCGGAAACCATGAAACCGATGGCTGTTCGTATGCACACCAATAACTTCAAAAAAGACCCGGAAACCCGTAAAACCTGCGTTGACTGCCACAAAGGTGTTGCGCACCCGTACCCGAAAGGTTAA

**Sequence A5: Codon optimized sequence of *stc*.**

ATGAGCAAAAAACTGCTGTCAGTTCTGTTCGGTGCTTCTCTGGCAGCGCTGGCACTGTCTCCGACCGCATTTGCAGCGGACCAGAAACTGAGCGACTTCCACGCTGAATCAGGTGGCTGCGAATCATGCCATAAAGACGGTACCCCGTCTGCAGACGGTGCCTTCGAATTCGCGCAGTGCCAGTCTTGCCACGGTAAACTGTCTGAAATGGACGCAGTTCACAAACCGCACGACGGTAACCTGGTTTGCGCTGACTGCCACGCTGTTCACGACATGAACGTTGGTCAGAAACCGACCTGCGAATCTTGCCACGACGACGGTCGTACCAGCGCATCTGTTCTGAAAAAATAA
